# A Novel Potentially Pathogenic Rare Variant in the *DNAJC7* Gene Identified in Amyotrophic Lateral Sclerosis Patients From Mainland China

**DOI:** 10.3389/fgene.2020.00821

**Published:** 2020-08-24

**Authors:** Mengli Wang, Zhen Liu, Yanchun Yuan, Jie Ni, Wanzhen Li, Yiting Hu, Pan Liu, Xiaorong Hou, Ling Huang, Bin Jiao, Lu Shen, Hong Jiang, Beisha Tang, Junling Wang

**Affiliations:** ^1^Department of Neurology, Xiangya Hospital, Central South University, Changsha, China; ^2^Department of Neurology, The Third Xiangya Hospital, Central South University, Changsha, China; ^3^Laboratory of Medical Genetics, Central South University, Changsha, China; ^4^Key Laboratory of Hunan Province in Neurodegenerative Disorders, Central South University, Changsha, China; ^5^National Clinical Research Center for Geriatric Diseases, Xiangya Hospital, Central South University, Changsha, China

**Keywords:** *DNAJC7*, amyotrophic lateral sclerosis, rare variant, heat-shock protein, Mainland China

## Abstract

Variants in the *DNAJC7* gene have been shown to be novel causes of amyotrophic lateral sclerosis (ALS). However, the contributions of *DNAJC7* mutations in Asian ALS patients remain unclear. In this study, we screened rare pathogenic variants in the *DNAJC7* gene in a cohort of 578 ALS patients from Mainland China. A novel, rare, putative pathogenic variant c.712A>G (p.R238G) was identified in one sporadic ALS patient. The carrier with this variant exhibited symptom onset at a relatively younger age and experienced rapid disease progression. Our results expand the pathogenic variant spectrum of *DNAJC7* and indicate that variants in the *DNAJC7* gene may also contribute to ALS in the Chinese population.

## Introduction

Amyotrophic lateral sclerosis (ALS) is an intractable neurodegenerative disorder characterized by progressive degeneration of the upper and lower motor neurons, leading to muscular weakness and atrophy. Most patients die from respiratory failure within 3–5 years of symptom onset (Chia et al., 2018). The number of ALS patients is rapidly increasing owing to the progressively aging population, with 400,000 patients projected to be diagnosed with ALS worldwide by 2040 (Arthur et al., 2016). Approximately 10% of ALS cases are familial ALS (fALS), with the remaining cases classified as sporadic ALS (sALS; Talbott et al., 2016). Genetic variation is an important risk factor for ALS. To date, mutations in more than 40 genes have been linked to the pathogenesis of ALS (Renton et al., 2014; Chia et al., 2018; Nicolas et al., 2018; Mathis et al., 2019; Li et al., 2020). These genetic discoveries have been essential in unraveling the molecular mechanisms underlying ALS (Chia et al., 2018).

Recently, Farhan et al. (2019) identified *DNAJC7*, a novel gene implicated in ALS, in a large, case-control, whole-exome sequencing (WES) study, in which they observed six distinct protein-truncating variants (PTVs) in eight individuals among 5,095 cases and none in 28,910 controls. Moreover, the authors also observed the depletion of DNAJC7 protein in fibroblasts from an ALS patient carrying truncation variant p.Arg156Ter of *DNAJC7*, which further validated the pathogenic role of *DNAJC7* mutations. In addition, they observed 15 rare missense variants in *DNAJC7*, of which four were predicted to be pathogenic in five ALS cases. However, this study did not include Asian patients, and the reported observations have not been validated by other studies. Previous studies have shown that genetic architectures differ among individuals of different ethnicities (Zou et al., 2017). For example, the hexanucleotide repeat expansion in the *C9ORF72* gene is the most frequent mutation in European ALS patients but is rare in Chinese and other Asian populations (Jiao et al., 2014; Liu et al., 2016).

In this study, owing to the ethnic heterogeneity of ALS-related genes and the relatively low number of genetic studies on *DNAJC7*, we investigated the potential contributions of *DNAJC7* variants to ALS in Mainland China.

## Materials and Methods

### Patients

We recruited a cohort of 578 ALS patients from Mainland China, including 535 patients with sALS and 43 probands with fALS. All patients were diagnosed with clinically definite, probable, or probable laboratory-supported ALS according to the revised EI Escorial criteria – 2015 (Ludolph et al., 2015). Clinical history acquisition, systematic physical examination, and evaluation of all patients were accomplished by at least two experienced neurologists. All patients provided written informed consent prior to participation in the study, and the study was approved by the Ethics Committee of Xiangya Hospital, Central South University.

### Mutation Analysis

#### Mutational Screening

Genomic DNA was extracted from the peripheral blood of each patient using standard protocols. WES was performed according to previously described methods (Wang et al., 2011; Zeng et al., 2018). All genome coordinates were based on the Genome Reference Consortium Human Build 37 (GRCh37). Variants with depth of coverage < 10, the allele balance < 0.25, or Phred quality score < 20 were excluded. Variants that met the following criteria were included for the further analysis: (1) being in the heterozygous state; (2) rarity, defined as a minor allele frequency (MAF) < 0.001 in the 1000 Genome Project (1000genome), the Exome Aggregation Consortium (ExAC), and Genome Aggregation Database (GnomAD); (3) exonic and non-synonymous, insertions, deletions, or variants predicted to affect splicing *in-silico*; and (4) pathogenicity, defined by at least five of 11 *in-silico* tools predicting pathogenicity (Quadri et al., 2018; Li et al., 2020). These criteria predicting variant pathogenicity was robust, as it takes into consideration that different *in-silico* tools were constructed based on different databases, algorithms, and focused on different aspects of pathogenic effects.

Sanger sequencing was performed to validate the putative pathogenic variants [c.712A>G (p.R238G)] using primers: F-CATCCTTGCAAAGCAGGAGGA, R-GGAAAACTGGCCACAAATGGT.

Patients carrying *DNAJC7* putative pathogenic variants were further screened for other ALS-related mutations. Nucleotide expansions in *C9ORF72* and *ATXN2* had also been excluded using a previously described method (DeJesus-Hernandez et al., 2011; Jiao et al., 2014).

#### Generation of a 3D Model of DNAJC7 Protein

Three-dimensional (3D) models of the DNAJC7 wild type and mutated protein were built using the SWISS-MODEL automated modeling server^1^, and 2y4t.1.A [Protein Data Bank (PDB) ID code] was used as the template. Models were visualized using Discovery Studio Visualizer software version 3.5 (Dassault Systèmes BIOVIA, San Diego, CA, USA).

DUET was used to assess protein stability. DUET consolidates two complementary approaches, Site Directed Mutator (SDM; Pandurangan et al., 2017) and mutation Cutoff Scanning Matrix (mCSM; Pires et al., 2014), to provide a consensus prediction by combining the results of the two separate methods with an optimized predictor.

## Results

### Mutation Analysis

Two novel rare missense variants in *DNAJC7*, c.712A>G (p.R238G) and c.281G>C (p.S94T), were identified in two unrelated sporadic ALS patients (Table 1). Variant c.712A>G (p.R238G) was absent in the ExAC, GnomAD, dbSNP, and 1000genome_Chinese online databases. Variant c.281G>C (p.S94T) was also absent in the 1000genome_Chinese database and had extremely low frequency in the ExAC and GnomAD databases. However, only variant c.712A>G (p.R238G) fulfilled the study’s pathogenicity criteria with eight out of 11 *in-silico* tools predicting pathogenicity (Tables 1 and 2).

**Table 1:** Rare variants in *DNAJC7* identified in the Chinese amyotrophic lateral sclerosis (ALS) cohort.

Chromosome position^a^	Exon	cDNA change	AA change	Mutation type	MAF GnomAD_ALL	MAF GnomAD_East Asian	MAF GnomAD_ non_neuro_ East Asian	MAF ExCA_ALL	MAF ExCA_ East Asian	1000genome_Chinese	dbSNP	Functional predictions: pathogenic (total)^b^
chr17:40141463	7	c.712A>G	p.R238G	missense	NA	NA	NA	NA	NA	NA	NA	8(11)
chr17:40149143	3	c.281G>C	p.S94T	missense	0.000812%	0.012%	0.0077%	0.000829%	0.01%	NA	rs782285650	1(11)

Putative pathogenic variant (black font); variant less likely to be pathogenic (gray font). ALS, amyotrophic lateral sclerosis; AA, amino acid; cDNA, complementary DNA; MAF, minor allele frequency; dbSNP, Database of Single Nucleotide Polymorphism; gnomAD, Genome Aggregation Database; ExAC, Exome Aggregation Consortium; non_neuro, population without neurological diseases; NA, not available.

a Position on Genome Reference Consortium Human Build 37 (GRCh37); NM_003315 was used for determining the nomenclature of *DNAJC7* variants.

b The *in-silico* tools and corresponding scores are listed in Table 2.

**Table 2:** Function prediction of the identified rare variants in *DNAJC7* by *in-silico* tools.

Variants	PolyPhen2 HDIV	PolyPhen2 HVAR	SIFT	PROVEAN	MetaLR	CADD	LRT	FATHMM	MCAP	Mutation taster	Mutation assessor
c.712A>G (p.R238G)	Possibly damaging (0.464)	Benign (0.284)	Damaging (0.023)	Damaging (−4.62)	Tolerable (0.237)	Damaging (23.8)	Deleterious(0.000)	Tolerate (−0.08)	Damaging (0.060)	Disease causing (1.000)	Medium (2.24)
c.281G>C (p.S94T)	Benign (0.0)	Benign (0.002)	Tolerable (0.795)	Tolerable (−0.48)	Tolerable (0.177)	Tolerable (15.05)	Neutral (0.011)	Tolerable (2.29)	Tolerable (0.010)	Disease causing (0.983)	Neutral (−1.305)

PolyPhen2 HDIV, Polymorphism Phenotyping Version 2 Human Diversity; PolyPhen2 HVAR, Polymorphism Phenotyping Version 2 Human Variation; SIFT, Sorting Intolerant From Tolerant; PROVEAN, Protein Variation Effect Analyzer; LR, logistic regression; CADD, combined annotation dependent depletion; LRT, likelihood ratio test; FATHMM, functional analysis through hidden Markov models; MCAP, Mendelian clinically applicable pathogenicity.

**Figure 1:**
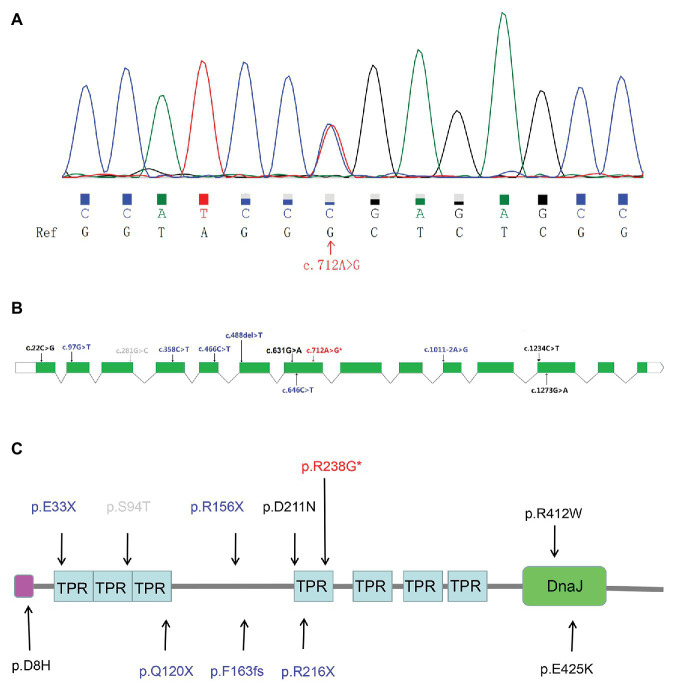
The Sanger sequencing and genetic locations of rare, putative pathogenic variants. **(A)** Sequence of the c.712A>G (p.R238G) variant sequence. Ref: reference sequence in NCBI. **(B)** Schematic representation of the *DNAJC7* transcript NM_003315. Previously reported rare, putative pathogenic missense variants (black font); previously reported rare, protein truncated variants (blue font); novel putative pathogenic variant (red font with an asterisk); and rare variant predicted to be benign (gray font). **(C)** Schematic representation of the DNAJC7 protein. Previously reported rare, putative pathogenic missense variants (black font); previously reported rare, protein truncated variants (blue font; the splicing variant is not shown because it affects non-coding regions of the gene); novel putative pathogenic variant (red font with an asterisk); and rare variant predicted to be benign (gray font). Pink block denotes low complexity region. TPR, tetratricopeptide repeat; DnaJ, DnaJ molecular chaperone homology domain.

Sanger sequencing further validated the heterozygous mutation c.712A>G (Figure 1A) in the ALS patient. Our screens for common ALS genes also excluded mutations in other ALS-related genes in the patient harboring variant c.712A>G (p.R238G). Interestingly, variant c.712A>G (p.R238G) discovered herein and two previously reported variants (c.631G>A and c.646C>T) were all found in exon 7 of *DNAJC7*. The locations of the two rare variants in our study and the previously reported rare, putative pathogenic variants are shown in Figures 1B,C.

**Figure 2:**
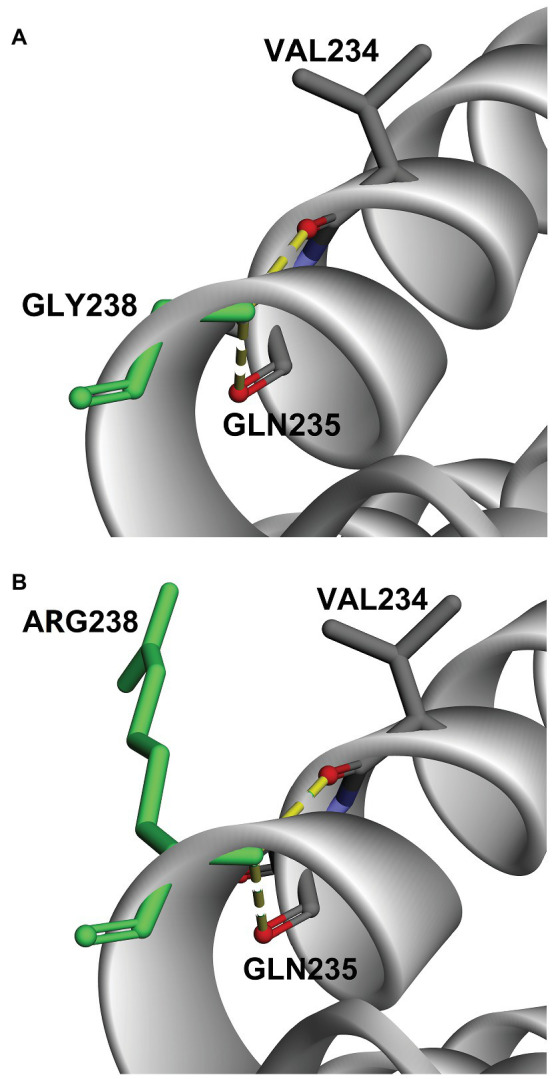
Comparison of 3D-model structures of **(A)** wild type DNAJC7 and **(B)** c.712A>G (p.R238G) variants.

To further understand the functional impact and pathogenic effect of the missense variant c.712A>G (p.R238G), we constructed 3D models of DNAJC7 wild type and mutated proteins, and assessed their stability. As shown in Figure 2, the amino acid at the 238th position converted from a positively charged amino acid (Arg) to Gly, which has no side chain. Interestingly, the hydrogen bond between site 238 and Val 234 or Gln 235 was not disrupted by changing Arg to Gly. However, DUET analysis predicted that mutation p.R238G would destabilize the DNAJC7 protein due to the negative free energy values (ΔΔG; Table 3).

**Table 3:** DUET stability of the identified *DNAJC7* putative pathogenic variant.

Variant	mCSM predicted stability change (ΔΔG)	SDM predicted stability change (ΔΔG)	DUET predicted stability change (ΔΔG)	Predicted stability change
c.712A>G (p.R238G)	−0.28 Kcal/mol	−0.85 Kcal/mol	−0.341Kcal/mol	decreased

mCSM, mutation Cutoff Scanning Matrix; SDM, site directed mutator.

### Clinical Features

The male patient carrying the heterozygous c.712A>G (p.R238G) variant initially suffered dysarthria, dysphagia, and muscle weakness in his upper limbs at 43 years of age. Four months later, his respiratory muscles were involved with mild dyspnea when he walked. Neurological examination revealed positive frontal release reflex on both sides, suggesting that the upper motor neurons may be affected. Muscle atrophy was obvious in all four extremities. Electromyography (EMG) revealed abundant and diffuse ongoing denervation (spontaneous potentials) and chronic reinnervation changes at four segments (bulbar, cervical, thoracic, and lumbar). No abnormalities were observed in brain magnetic resonance imaging (MRI). The patient’s ALS functional rating scale revised (ALSFRS-R) score was 39 out of 48. Additionally, no cognitive impairment was indicated in the Mini-Mental State Examination. The patient reported a negative family history of any neurological disease. The patient eventually died of respiratory failure 1 year after disease onset.

## Discussion

DNAJC7 belongs to the DnaJ heat shock protein family (Hsp40), which assists a wide range of folding processes, such as folding newly synthesized polypeptides and clearing degraded proteins (Jiang et al., 2007). DNAJ proteins act as co-chaperones for HSP70 proteins and constitute a complex network of the folding machinery with HSP70 (Mayer and Bukau, 2005; Jiang et al., 2007). Abnormal expression of *HSP70* and *DNAJ* genes affects the network dynamics and causes the formation of protein aggregates, which are a critical feature of many neurodegenerative diseases such as Alzheimer’s disease, Parkinson’s disease, Huntington’s disease, prion disease, and ALS (Lackie et al., 2017). Recently, Farhan et al. (2019) identified *DNAJC7* as a novel ALS risk gene in a large case-control exome sequencing study and demonstrated the function loss of *DNAJC7* PTV (p.Arg156Ter) by functional validation. In their study, six distinct, rare PTVs in eight ALS patients and four rare, putative pathogenic missense mutations in five ALS cases were observed among 5,095 ALS patients; none of these variants were observed among 28,910 controls. Association analysis revealed that PTV variants may confer risk for ALS. However, Asian patients were not recruited in the study. Therefore, we sought to investigate *DNAJC7* variants in the Chinese ALS population and validate whether *DNAJC7* is a novel ALS risk gene among this population.

In this study, we identified a novel rare missense variant c.712A>G (p.R238G) in the *DNAJC7* gene in one sporadic ALS patient. Eight out of 11 *in-silico* tools predicted this variant to be pathogenic. Three-dimensional models of wild type and mutated DNAJC7 proteins and DUET analysis suggested that the substitution of Arg to Gly in the mutated protein could decrease its stability. However, further functional studies are warranted to validate the pathogenicity of this variant. In contrast, another rare variant identified in our study, c.281G>C (p.S94T), was predicted to be tolerated and benign by 10 out of 11 *in-silico* tools, likely because Ser and Thr share similar structures and polarity.

The frequency of *DNAJC7* putative pathogenic variants carried in ALS patients in our study (0.17%) was lower than that in white populations (0.25%; Farhan et al., 2019). The discrepancy in mutation sites and prevalence of *DNAJC7* putative pathogenic mutations between these two studies may arise from the different ethnic backgrounds of the patients. Previous studies also reported differences in the prevalence rates of mutations in other ALS-related genes, such as *C9ORF72*, *SOD1*, and *TIA1*, across different ethnicities (Jiao et al., 2014; Liu et al., 2016; Yuan et al., 2018).

No studies have reported clinical phenotypes associated with *DNAJC7* mutations. In this study, the carrier with the c.A712G (p.R238G) variant presented with initial symptoms in the spinal and bulbar regions. The patient also experienced onset at a relatively younger age (43 years) and rapid disease progression (respiratory muscles involved within 4 months after symptom onset, died of respiratory failure within 1 year after symptom onset). Thus, the c.A712G (p.R238G) variant may be associated with a rapidly progressive course and a worse prognosis. Nevertheless, there was only one patient carrying this variant in our study. More robust independent studies are warranted to confirm the relationships between ALS-related *DNAJC7* variants and clinical phenotypes.

In summary, we first screened *DNAJC7* variants in a large cohort of Chinese ALS patients and identified a novel, putative pathogenic variant c.A712G (p.R238G) in one sALS patient. Including the rare, putative pathogenic variant identified in our study, only 11 *DNAJC7* putative pathogenic variants have been reported in ALS patients. Although the prevalence of *DNAJC7* pathogenic mutations varies among different ethnic populations, identification of a novel variant in our study suggests that *DNAJC7* may also play an important role in Chinese patients with ALS. Moreover, the c.A712G (p.R238G) variant may be a predictor of early onset and poor prognosis. Additional studies with larger sample sizes are warranted to elucidate the potential contribution of *DNAJC7* variants to ALS.

## Data Availability Statement

According to national legislation/guidelines, specifically the Administrative Regulations of the People’s Republic of China on Human Genetic Resources (http://www.gov.cn/zhengce/content/2019-06/10/content_5398829.htm, http://english.www.gov.cn/policies/latest_releases/2019/06/10/content_281476708945462.htm), no additional data are available at this time. Data of this project can be accessed after an approval application to the China National Genebank (CNGB, https://db.cngb.org/cnsa/“target=“_blank”>https://db.cngb.org/cnsa/“target=“_blank”>https://db.cngb.org/cnsa/“target=“_blank”>https://db.cngb.org/cnsa/). Please refer to https://db.cngb.org/, or email: CNGBdb@cngb.org for detailed application guidance. The accession code CNP0001152 should be included in the application.

## Ethics Statement

All patients provided written informed consent for the genetic research, and this study was approved by the Ethics Committee of Xiangya Hospital, Central South University.

## Author Contributions

MW analyzed data and wrote original draft. ZL, YY, JN, WL, YH, PL, XH, LH, and BJ collected clinical data. LS, HJ, and BT supervised the process. JW designed the study and edited the manuscript. All authors contributed to the article and approved the submitted version.

### Conflict of Interest

The authors declare that the research was conducted in the absence of any commercial or financial relationships that could be construed as a potential conflict of interest.

## Footnotes

^1^https://swissmodel.expasy.org/interactive
